# Electric Transport in Few-Layer ReSe_2_ Transistors Modulated by Air Pressure and Light

**DOI:** 10.3390/nano12111886

**Published:** 2022-05-31

**Authors:** Enver Faella, Kimberly Intonti, Loredana Viscardi, Filippo Giubileo, Arun Kumar, Hoi Tung Lam, Konstantinos Anastasiou, Monica F. Craciun, Saverio Russo, Antonio Di Bartolomeo

**Affiliations:** 1 Department of Physics “E.R. Caianiello”, University of Salerno, 84084 Fisciano, SA, Italy; efaella@unisa.it (E.F.); k.intonti@studenti.unisa.it (K.I.); l.viscardi7@studenti.unisa.it (L.V.); akumar@unisa.it (A.K.); 2 CNR-SPIN, 84084 Fisciano, SA, Italy; filippo.giubileo@spin.cnr.it; 3 University of Exeter, Stocker Road 6, Exeter EX4 4QL, Devon, UK; o.lam@exeter.ac.uk (H.T.L.); ka391@exeter.ac.uk (K.A.); m.f.craciun@exeter.ac.uk (M.F.C.); s.russo@exeter.ac.uk (S.R.)

**Keywords:** 2D materials, rhenium, selenides, ReSe_2_, field-effect transistor, pressure, negative photoconductivity

## Abstract

We report the fabrication and optoelectronic characterization of field-effect transistors (FETs) based on few-layer ReSe_2_. The devices show n-type conduction due to the Cr contacts that form low Schottky barriers with the ReSe_2_ nanosheet. We show that the optoelectronic performance of these FETs is strongly affected by air pressure, and it undergoes a dramatic increase in conductivity when the pressure is lowered below the atmospheric one. Surface-adsorbed oxygen and water molecules are very effective in doping ReSe_2_; hence, FETs based on this two-dimensional (2D) semiconductor can be used as an effective air pressure gauge. Finally, we report negative photoconductivity in the ReSe_2_ channel that we attribute to a back-gate-dependent trapping of the photo-excited charges.

## 1. Introduction

Rhenium diselenide (ReSe_2_) is a member of the layered transition metal dichalcogenides (TMDs), which has attracted a lot of attention due to the extremely anisotropic electrical, optical and mechanical properties stemming from the strong in-plane anisotropy consequence of its reduced crystal symmetry [1,2,3,4]. Contrary to other hexagonal TMDs, the room temperature thermodynamically stable 1T phase for ReSe_2_ has a distorted triclinic symmetry, which endows the material with anisotropic responses in many properties [5,6,7].

Monolayer ReSe_2_ has an indirect bandgap of 1.34 eV [8,9,10], reducing to 0.98 eV [6] for bulk ReSe_2,_ with a weak layer dependency. In general, an increase in the layer thickness causes a reduction in band-gap energy and the loss of electric properties of thick ReSe_2_ [11].

ReSe_2_ has been employed in various electronic and optoelectronic functional devices in order to study its electrical and optical properties. Yang et al. reported that the mobility of ReSe_2_ nanosheets increases when the number of layers decreases and highlighted that the properties of ReSe_2_ can be tuned by the number of layers and gas molecule gating, making ReSe_2_ a promising material for future functional device applications [11]. Optically biaxial and highly anisotropic Mo-doped ReSe_2_ (Mo:ReSe_2_) was used to investigate the effects of physisorption of gas molecules on few-layer nanosheet-based photodetectors, reporting different sensitivity to the surrounding environment, prompt photoswitching, and high photoresponsivity [12].

The anisotropic nature of ReSe_2_ was revealed by Raman spectroscopy under linearly polarized excitations in a study by Zhang et al., who fabricated top-gate ReSe_2_ field-effect transistors (FETs), with a high on/off current ratio and a well-developed current saturation in the current–voltage characteristics at room temperature [7]. They synthesized ReSe_2_ directly onto hexagonal boron nitride (h-BN) substrates to improve the electron and hole mobility and demonstrated that the ReSe_2_-based photodetectors exhibit polarization-sensitive photoresponsivity due to the intrinsic linear dichroism, originating from high in-plane optical anisotropy, thus, identifying ReSe_2_ as a highly anisotropic two-dimensional (2D) material for novel electronic and optoelectronic applications.

Similarly, a near-infrared ReSe_2_ photodetector featuring high photoresponsivity and a short photoresponse time, in the order of 10 ms, was demonstrated by Kim and coworkers, achieving high photo and temporal responses simultaneously by applying a p-doping technique based on hydrochloric acid to a selected ReSe_2_ region [13].

Ambipolar FETs were obtained from multi-layer ReSe_2_, mechanically exfoliated onto a SiO_2_ layer by Pradhan et al., who demonstrated that it is possible to utilize the ambipolarity to fabricate logical elements or digital synthesizers [10]. Similarly, ambipolar all-2D ReSe_2_ FET with a h-BN gate dielectric and graphene contacts were investigated by Lee and coworkers, who used the ambipolar transfer characteristics, attributed to the tunable Fermi level of the graphene contact, to demonstrate an inverter in a logic circuit [14].

Corbet et al. proposed a method to improve the contact resistance in few-layer ReSe_2_ FETs, by up to three orders of magnitude, using ultra-high-vacuum annealing [15]. A low contact resistance was also obtained in single-layer ReSe_2,_ encapsulated in h-BN using scandium/gold contacts, and this enabled Khan and coworkers [16] to measure a large field-effect charge carrier mobility and responsivity.

Xing et al. addressed the challenge of the controlled synthesis of high-quality ultrathin ReSe_2_, developing an approach for synthesizing 2D ReSe_2_ flakes with a thickness down to monolayer by chemical vapor transport, through carefully tuning the growth kinetics [17]. The FETs fabricated with such flakes showed n-type semiconducting behavior with mobility of a few cm^2^ V^−1^ s^−1^, comparable to the values measured using mechanically exfoliated flakes.

Polarization-resolved ReSe_2_ photodetectors were recently studied by Tian and Liu, who reported a van der Waals heterojunction ReSe_2_/WSe_2_-based photodetector, with high responsivity and detectivity at room temperature. Remarkably, they demonstrated that the photoresponse of their devices is a function of the polarized angle of the incident light, indicating the effective polarized light detection [18].

Pressure is commonly used to understand the interlayer interaction in layered materials. High-hydrostatic pressures of several kbar were applied to ReSe_2_ (and ReS_2_) exfoliated flakes and the effect on their optical properties was investigated, finding that the energies of the two main excitonic transitions decrease in energy with increasing pressure [19]. The negative pressure coefficients were attributed to the destabilization of the p_z_ orbital with increasing pressure, demonstrating that ReSe_2_ does not exhibit a strong electronic decoupling and, hence, the optoelectronic properties of few-layered ReSe_2_ could be drastically different from the bulk form.

Conversely, the effect of low pressure on ReSe_2_ has been rarely investigated in the literature.

In the present study, we fabricate back-gate FETs with a few-layer ReSe_2_ channel and study the electric transport from room pressure down to 10^−5^ mbar. We find that air pressure has a dramatic effect on the channel conductivity, which increases by more than two orders of magnitude when the pressure decreases. We explain such behavior in terms of the desorption of oxygen and water molecules from the ReSe_2_ surface in high vacuum. Importantly, we observe that the effect of air pressure is reversible, highlighting that back-gate ReSe_2_ FETs can be exploited as effective pressure gauges. Moreover, we report a reduction of the channel conductivity when the device is illuminated, i.e., a negative photoconducticity, that has not been reported before for ReSe_2_. The dependence of the negative photoconductivity on the gate voltage suggests that photo-excited free charge carriers are attracted towards the gate and captured at the interface, with the dielectric layer contributing to the observed loss of conductivity.

## 2. Materials and Methods

**Figure 1:**
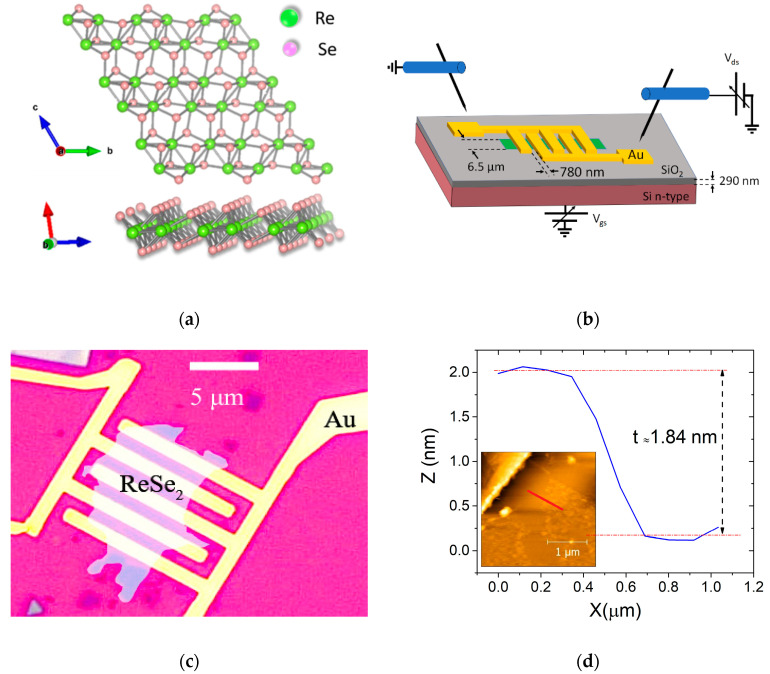
(**a**) Top view and side view of ReSe_2_ atomic structure (the green and pink dots represent the Re and Se atoms, respectively); (**b**) schematic of the ReSe_2_ back-gated FET with interdigitated source/drain leads. (**c**) Optical image of the ReSe_2_ device with interdigitated Cr/Au leads. The flake is highlighted. (**d**) AFM vertical profile showing the flake thickness of 1.84 nm.

Ultrathin ReSe_2_ flakes were exfoliated from bulk ReSe_2_ single crystals using a standard mechanical exfoliation method by adhesive tape. The flakes were transferred onto highly doped n-type (resistivity 0.005 Ω cm) silicon substrates, covered by 290 nm thick SiO_2_, which serves as a global back gate. Photolithography and standard lift-off process of evaporated Cr/Au (5 nm/100 nm) were applied to define metal contacts. Figure 1a reports the crystal structure and Figure 1b shows the schematic of a ReSe_2_ FET with the circuit used to control the Si/SiO_2_ back-gate and the source-drain bias on the 2D semiconducting channel. We adopted an interdigitated layout with 4 parallel channels corresponding to a total channel width W = 26.0 μm and length L = 0.78 μm. An optical top view of a typical device is shown in Figure 1c. The thickness of the flake was measured by an atomic force microscope (Nanosurf AG, Liestal, Switzerland), obtaining the height profile displayed in Figure 1d that confirms a thickness of 1.84 nm, corresponding to 3 layers [20].

Electric measurements were carried out in two-probe configuration in a Janis ST-500 Probe Station (Lake Shore Cryotronics, Inc., Westerville, OH, USA) equipped with nanoprobes connected to the source/drain leads (Figure 1b). The back-gate voltage was applied through the sample holder of the probe station which was in direct electrical contact to the Ag-pasted n-Si substrate. The measurements were performed by the source-measurement units of a semiconductor characterization system Keithley 4200 SCS (Tektronix, Inc., Beaverton, OR, USA), with current and voltage sensitivity better than 1 pA and 2 μV, respectively. For the transistor characterization, the source was grounded while the drain ($V_{\mathrm{ds}}$) and gate ($V_{\mathrm{gs}}$) voltages were either swept or stepped while the drain ($I_{d}$) and gate ($I_{g}$) currents were monitored. The measured gate leakage current was always < 10 pA, confirming the integrity of the SiO_2_ gate dielectric.

The electric measurements were performed at controlled air pressure, from room pressure to 10^−5^ mbar. Under the combined action of a rotatory and a turbomolecular pump connected in series to a probe station and a valve system, it was possible to control the pressure stepwise. The pressure was monitored through the pressure gauge TPG261 (Pfeiffer, Asslar, Deutschland). The photoresponse of the device was investigated using an array of 144 white LEDs with a spectrum ranging from 400 to 750 nm and peaks at 450 nm and 540 nm, a color temperature of 6000 K, and with 1 mW/cm^2^ intensity.

## 3. Results and Discussion

Initially, the ReSe_2_ transistor was characterized in dark and at room temperature and pressure, followed by investigating the effect of the lowering pressure in the same conditions of temperature and darkness. Finally, we explored the photoresponse of the fabricated device.

### 3.1. Transistor Characterization

**Figure 2:**
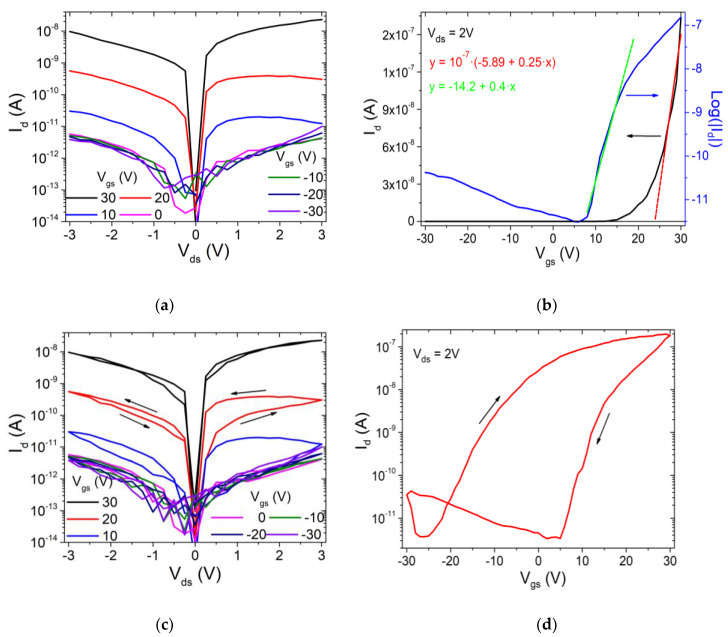
Electrical measurements at normal atmospheric pressure: (**a**) Output curves for reverse $\;V_{\mathrm{ds}}\;$ sweep (single). (**b**) Transfer curve on linear (black) and logarithmic (blue) scale. (**c**) Output curves for forward and reverse $V_{\mathrm{ds}}$ sweeps. (**d**) Transfer curves for forward and reverse $V_{\mathrm{gs}}\;$ sweeps, showing a wide hysteresis.

Figure 2a,b report the output ($I_{d}-V_{\mathrm{ds}}$ at fixed $V_{\mathrm{gs}}$) and transfer ($I_{d}-V_{\mathrm{gs}}$ at fixed $V_{\mathrm{ds}}$) characteristics of the fabricated ReSe_2_ FET, respectively. We limited the drain bias to 3 V and gate voltage range to ± 30 V to prevent damage to the device and, in particular, to the SiO_2_ gate dielectric. The $I_{d}-V_{\mathrm{ds}}$ curves (Figure 2a) show that the drain current is modulated by the gate voltage $V_{\mathrm{gs}}$ and stays below 10 pA for negative $V_{\mathrm{gs}}$ but increases abruptly for positive $V_{\mathrm{gs}}$. This behavior is typical of a n-type transistor [21,22]. Furthermore, for all gate voltages, the $I_{d}-V_{\mathrm{ds}}$ curves are asymmetric, with slightly higher current at positive $V_{\mathrm{ds}}$, pointing to the formation of low Schottky barriers at the ReSe_2_/Cr/Au contacts [23,24,25,26]. The presence of a Schottky barrier is confirmed also by the limited current that reaches the maximum of 20 nA at $V_{\mathrm{ds}}=3\;V$.

The $I_{d}-V_{\mathrm{gs}}$ transfer curves of Figure 2b, shown on both the linear and logarithmic scale, confirm the n-type behavior of the transistor, with off-state at $V_{\mathrm{gs}}<20\;V$ and on-state for $V_{\mathrm{gs}}>20\;V$. The curve on the logarithmic scale shows an on/off current ratio higher than two orders of magnitude and a modest subthreshold swing SS≃2.8 V/decade, typical of back-gate 2D transistors with limited gate efficiency and high interface defect density [27,28,29,30]. The smooth rise of $I_{d}$ at negative $V_{\mathrm{gs}}$ indicates the appearance of a hole-type conduction. The carrier type can be controlled via the metal contacts. Dominant n-type behavior is obtained in ReSe_2_ transistors with low-work-function metal contacts, such as Al or Ti, whose Fermi level aligns above the conduction band minimum of ReSe_2_ [7,14,31]. As the conduction band minimum of ReSe_2_ is around of 4.5 eV and the valence band maximum is around 5.6 eV [31], the Fermi levels of Cr and Au that have work functions of 4.5 and 5.1 eV, respectively, align within the ReSe_2_ bandgap and can favor ambipolar conduction.

The transfer curve on the linear scale is used to estimate the field-effect mobility, $\mu_{\mathrm{FE}}$, in the on-state of the transistor for $V_{\mathrm{gs}}>20\;V$. The mobility, evaluated as $\mu_{\mathrm{FE}}=\frac{L}{W}\frac{1}{C_{\mathrm{ox}}V_{\mathrm{ds}}}\frac{{\mathrm{dI}}_{\mathrm{ds}}}{{\mathrm{dV}}_{\mathrm{gs}}}$ (here $C_{\mathrm{ox}}=1.15\times 10^{-8\;}{F\;\mathrm{cm}}^{-2}$ is the gate dielectric capacitance per unit area), results $\mu_{\mathrm{FE}}≃0.03{\;\mathrm{cm}}^{2\;}V^{-1}{\;s}^{-1}$ slightly lower than the $\mu_{\mathrm{FE}}\;∼\;0.1-10{\;\mathrm{cm}}^{2}\;V^{-1}{\;s}^{-1}$, typically measured in few-layer ReSe_2_ FETs [5,7,10,11]. We also note that an increase in layer thickness causes a loss of electric properties in ReSe_2_ and, in particular, that few-layer ReSe_2_ exhibits lower mobility of two orders of magnitude or more than single-layer ReSe_2_ [11]. Furthermore, the presence of a Schottky barrier at the contacts [32,33], as well as intrinsic defects in the material and impurities located at the interface with the SiO_2_ layer or adsorbates on top of the channel from air exposure during the fabrication and the measurement process [10,34,35], acting as scattering or trapping centers, can contribute to decrease the mobility.

The x-axis intercept of the straight line that fits the transfer curve on the linear scale in Figure 2b is assumed as the threshold voltage $V_{\mathrm{th}}$ of the transistor and is about 20 V, indicating a n-type enhancement mode device.

More insights in the electric transport through the ReSe_2_ channel can be gained from Figure 2c,d, which display a hysteresis on both the output and transfer curves when $V_{\mathrm{ds}}$ or $V_{\mathrm{gs}}$ are swept in a loop (the forward and reverse sweeps yield different curves). The presence of large hysteresis in the $I_{d}-V_{\mathrm{ds}}$ characteristics has been reported before in monolayer MoS_2_ devices, where it was attributed to the multigrain structure of the material and exploited to enable resistive switching devices. The presence of grain boundaries provides the opportunity to fabricate memristors, owing to the phenomenon of migration of defects, such as sulphur vacancies at grain boundaries, by applying a high electric field [36]. The hysteretic behavior in Figure 2c points to a defective ReSe_2_ channel, possibly with Se vacancies, consistent with the n-type intrinsic doping and the low mobility. The presence of intrinsic and interfacial defects is confirmed by the huge hysteresis observed in the transfer curve in Figure 2d. Hysteresis in the transfer characteristic is very common in 2D-material-based transistors and has been widely studied and attributed to charge trapping inside the channel material, interface trap states or surface adsorbates [37,38]. The interaction with the SiO_2_ dielectric, i.e., the ReSe_2_/SiO_2_ interface, is of paramount importance. Indeed, the substitution of the SiO_2_ layer by a high-quality h-BN-insulating substrate, which is atomically flat and free of charge trapping sites, has been shown to result in a strong mitigation of the hysteresis [39].

### 3.2. Pressure Behavior

**Figure 3:**
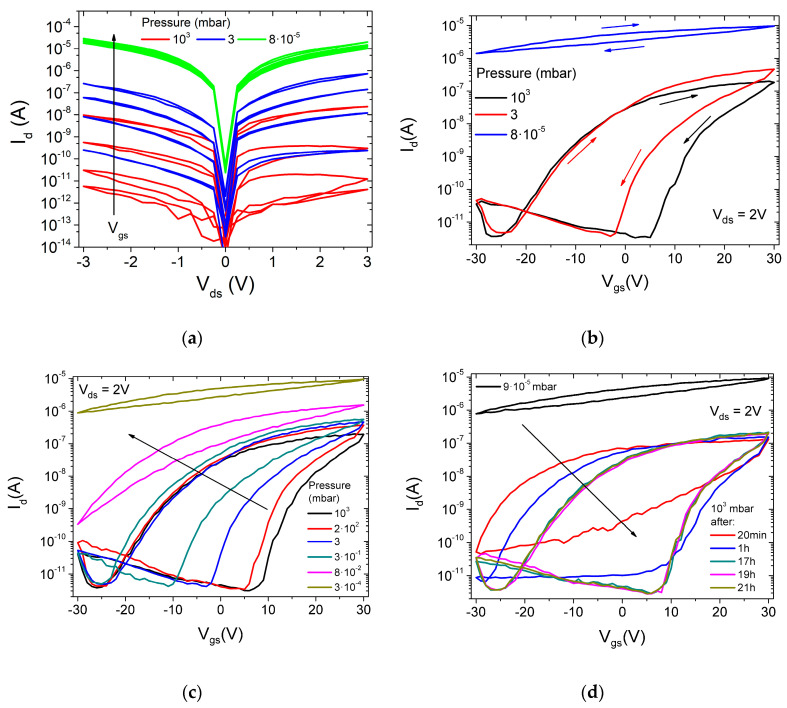
(**a**) Output curves at different gate voltages ($V_{\mathrm{gs}}$ = 0, 10, 20, 30 V) for three different air pressures (atmospheric, 3 mbar, $8\times 10^{-5}$ mbar). (**b**) Transfer curves for three different air pressures (atmospheric, 3 mbar, $8\times 10^{-5}$ mbar). The arrows show the direction of voltage gate sweeping starting from 30 V. Transfer characteristics (**c**) for lowering pressure, and (**d**) at different times after reaching the room pressure.

To investigate the effect of air pressure on the ReSe_2_ channel conductivity, we performed an electric transport measurement, lowering the atmospheric pressure down to 10^−5^ mbar. The measurements were performed after keeping the device at the given pressure for several hours to achieve a steady state. Figure 3a shows the output characteristics at three different pressures (room pressure, 3 mbar and $8\times 10^{-5}$ mbar) for increasing gate voltages, ranging from 0 V to 30 V, with steps of 10 V. It can be observed that the channel current increases at lower pressure while the hysteresis decreases, and the asymmetric behavior is unchanged. The reduced hysteresis indicates that surface adsorbates play an important role.

The same trend with increased current and reduced hysteresis at low pressure is displayed also by the transfer characteristics in Figure 3b. The low pressure, in particular, causes a dramatic change in the transfer characteristics with the transistor that does not turn off over the applied $V_{\mathrm{gs}}$ range. The lowering pressure causes a left shift in the transfer characteristics, corresponding to a reduction in the threshold voltage $V_{\mathrm{th}}$, pointing to an increased n-type doping density. Such behavior can be explained as desorption of adsorbates from the ReSe_2_ surface. Adsorbed oxygen and water molecules, being electronegative, subtract electrons to the channel, thus, decreasing the conductivity (otherwise stated, oxygen and water counter-dope the n-type channel with holes). Their desorption has the two-fold beneficial effect of increasing the n-type doping and the mobility (see following), resulting in increased conductivity.

Figure 3c,d, which display the transfer characteristics for lowering and raising pressures, respectively, demonstrate that the transformation of the transfer curves is gradual and reversible. While the plot in Figure 3c shows the dynamic evolution of the transfer curves during the pressure change, the plot in Figure 3d monitors the time evolution of the transfer curves after a sudden change from $8\times 10^{-5}$ mbar to room pressure, showing that the recovery of the pristine state is a slow process, requiring a few hours. The reversible change of current with pressure demonstrates that the device can be used as an air pressure gauge.

**Figure 4:**
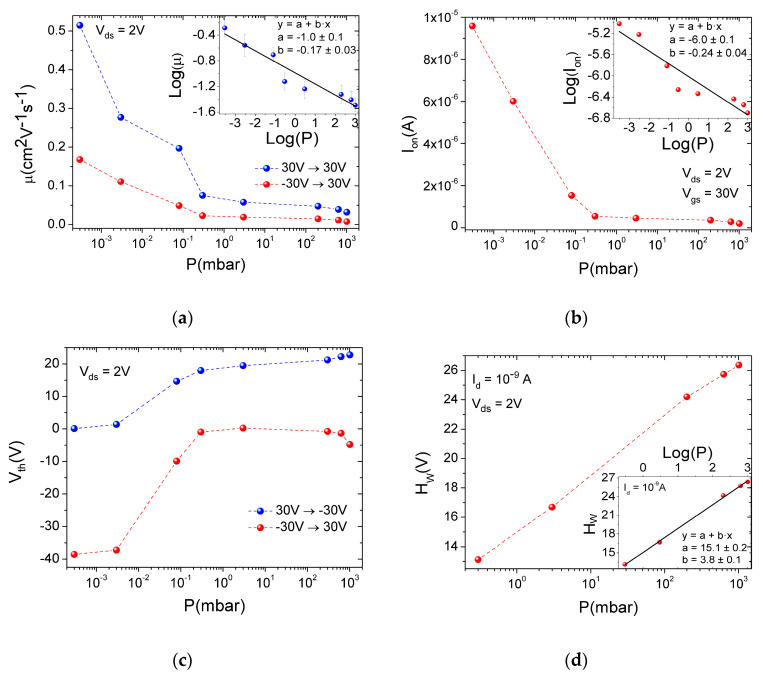
(**a**) Forward and reverse mobility as function of pressure. Linear fit of the data on log–log scale in the inset, (**b**) current in the on state as function of pressure. Linear fit of the data on log–log scale in inset, (**c**) forward and reverse threshold voltage as function of pressure, and (**d**) hysteresis width at $I_{d}$ = 1 nA versus air pressure. Linear fit of data on semi-log scale in the inset.

Figure 4a,b detail the behavior of the mobility $\mu_{\mathrm{FE}}$ and of the current in the on state ($I_{\mathrm{on}}$) as a function of pressure. The mobility was evaluated using both the forward ($V_{\mathrm{gs}}$ sweep from −30 V to 30 V) and reverse ($V_{\mathrm{gs}}$ sweep from 30 V to −30 V) branches of transfer characteristics. Both forward $\mu_{\mathrm{FE}}$ and $I_{\mathrm{on}}$ decrease for increasing pressure, following a power law, as demonstrated by the linear log–log plots in the respective insets. Conversely, the threshold voltage $V_{\mathrm{th}}$ increases up to $10^{-1}$ mbar, above which it reaches a plateau (Figure 4c), demonstrating that the desorption of the adsorbates becomes effective at a pressure below $10^{-1}$ mbar. Finally, Figure 4d shows that the hysteresis width (here defined as the difference between the $V_{\mathrm{gs}}$ corresponding to the current $I_{d}$ = 1 nA in the reverse and forward sweep) is also increased by the rising pressure. The contribution of adsorbates to hysteresis in 2D-material-based transistors has been widely studied and demonstrated [34,40,41]. The easier the charge transfer between the channel and the adsorbates, the wider the hysteresis [38].

### 3.3. Photoresponse

As ReSe_2_ nanosheets have been widely used in efficient photodetectors [7,13,42], we checked the photoresponse of the ReSe_2_ FET by exposing it to the light of an array of white LEDs at a pressure of $8\times 10^{-5}$ mbar.

**Figure 5:**
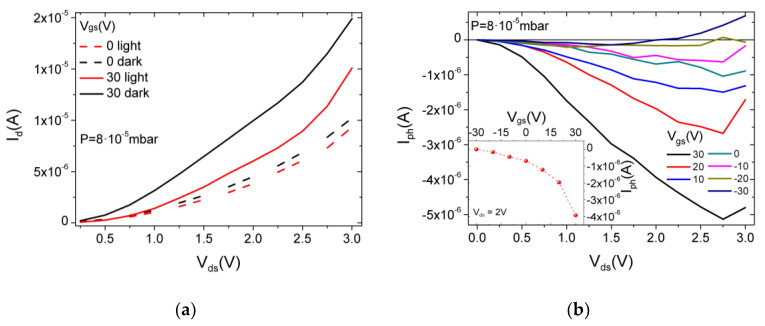
(**a**) $I_{\mathrm{ds}}-V_{\mathrm{ds}}$ curves in dark and light at $V_{\mathrm{gs}}=$ 0 and +30 V (dashed and solid lines); (**b**) $I_{\mathrm{ph}}$ − $V_{\mathrm{ds}}$ curves at different $V_{\mathrm{gs}}$. $I_{\mathrm{ph}}$ vs. $V_{\mathrm{gs}}$ at $V_{\mathrm{ds}}$ = 2 V in the inset.

Figure 5a shows that the current $I_{d}$ decreases when the device is illuminated, a phenomenon referred to as negative photoconductivity. The decrease in the current under light is enhanced at $V_{\mathrm{gs}}$ = 30 V. Illumination normally generates additional carriers in a semiconductor material, which increase its conductivity. Conversely, negative photoconductivity has been reported in a few 1D and 2D materials, and explained as a photogating effect due to trap centers, light-induced desorption of surface gas molecules or surface plasmons [43,44,45,46,47]. The origin and role of the negative photoconductivity in low-dimensional materials is still poorly understood. Moreover, negative photoconductivity has not been observed before in ReSe_2_ and requires deep investigation that will be the subject of a forthcoming study. Here, we note that the photocurrent ($I_{\mathrm{ph}}=I_{\mathrm{light}}-I_{\mathrm{dark}}$) increases with the drain bias and has the absolute value tunable by the gate voltage, as shown in Figure 5b. The increase in the photocurrent with $V_{\mathrm{ds}}$ is easily understood because a higher horizontal field favors charge collection to the drain. The increasing $\left|I_{\mathrm{ph}}\right|$ with the higher gate bias instead suggests a mechanism for the negative photoconductivity, as gate-induced photo-excited charges separation and trapping. The photogenerated electron-hole pairs are separated by the vertical gate field, which attracts electrons at the ReSe_2_/SiO_2_ interface, where they become trapped. The excess holes in the channels combine with electrons of the n-type ReSe_2_, causing a counter-doping effect, i.e., a reduction in the channel conductivity.

## 4. Conclusions

We fabricated a back-gate field-effect transistor with ReSe_2_ nanosheets and Cr/Au contacts and studied its electric transport. We showed that the transistor has a dominant n-type character due to the alignment of the Cr Fermi level with the ReSe_2_ conduction band minimum. We investigated the effect of low pressure on the material conductivity and found that the device is strongly affected by air pressure. The exposure to air suppresses the channel conductivity as an effect of electron capture by oxygen and water molecules adsorbed on the material surface. The desorption of adsorbates in high vacuum increases the channel conductivity. We pointed out that the reversible pressure behavior allows the device to be used as an air pressure gauge. Furthermore, we showed that the n-type channel and the gate-driven separation and trapping of photogenerated electrons can lower the channel conductivity under illumination, the origin of the observed negative photoconductivity.

## Data Availability

The data presented in this study are available on request from the corresponding author.

## References

[1] Friemelt K., Lux-Steiner M.C., Bucher E. (1993). Optical Properties of the Layered Transition-Metal-Dichalcogenide ReS_2_: Anisotropy in the van Der Waals Plane. J. Appl. Phys..

[2] Ho C.H., Huang C.E. (2004). Optical Property of the Near Band-Edge Transitions in Rhenium Disulfide and Diselenide. J. Alloys Compd..

[3] Dumcenco D.O., Huang W.Y., Huang Y.S., Tiong K.K. (2009). Anisotropic Optical Characteristics of Au-Doped Rhenium Diselenide Single Crystals. J. Alloys Compd..

[4] Di Bartolomeo A. (2020). Emerging 2D Materials and Their Van Der Waals Heterostructures. Nanomaterials.

[5] Cui F., Li X., Feng Q., Yin J., Zhou L., Liu D., Liu K., He X., Liang X., Liu S. (2017). Epitaxial Growth of Large-Area and Highly Crystalline Anisotropic ReSe_2_ Atomic Layer. Nano Res..

[6] Ho C.H., Huang Y.S., Tiong K.K. (2001). In-Plane Anisotropy of the Optical and Electrical Properties of ReS_2_ and ReSe_2_ Layered Crystals. J. Alloy. Compd..

[7] Zhang E., Wang P., Li Z., Wang H., Song C., Huang C., Chen Z.-G., Yang L., Zhang K., Lu S. (2016). Tunable Ambipolar Polarization-Sensitive Photodetectors Based on High-Anisotropy ReSe_2_ Nanosheets. ACS Nano.

[8] Wolverson D., Crampin S., Kazemi A.S., Ilie A., Bending S.J. (2014). Raman Spectra of Monolayer, Few-Layer, and Bulk ReSe_2_: An Anisotropic Layered Semiconductor. ACS Nano.

[9] Hart L.S., Webb J.L., Dale S., Bending S.J., Mucha-Kruczynski M., Wolverson D., Chen C., Avila J., Asensio M.C. (2017). Electronic Bandstructure and van Der Waals Coupling of ReSe_2_ Revealed by High-Resolution Angle-Resolved Photoemission Spectroscopy. Sci. Rep..

[10] Pradhan N.R., Garcia C., Isenberg B., Rhodes D., Feng S., Memaran S., Xin Y., McCreary A., Walker A.R.H., Raeliarijaona A. (2018). Phase Modulators Based on High Mobility Ambipolar ReSe_2_ Field-Effect Transistors. Sci. Rep..

[11] Yang S., Tongay S., Li Y., Yue Q., Xia J.-B., Li S.-S., Li J., Wei S.-H. (2014). Layer-Dependent Electrical and Optoelectronic Responses of ReSe_2_ Nanosheet Transistors. Nanoscale.

[12] Yang S., Tongay S., Yue Q., Li Y., Li B., Lu F. (2015). High-Performance Few-Layer Mo-Doped ReSe_2_ Nanosheet Photodetectors. Sci. Rep..

[13] Kim J., Heo K., Kang D., Shin C., Lee S., Yu H., Park J. (2019). Rhenium Diselenide (ReSe_2_) Near-Infrared Photodetector: Performance Enhancement by Selective P-Doping Technique. Adv. Sci..

[14] Lee K., Yang S., Sung Y., Chang Y., Lin C., Yang F., Li M., Watanabe K., Taniguchi T., Ho C. (2019). Analog Circuit Applications Based on All-2D Ambipolar ReSe_2_ Field-Effect Transistors. Adv. Funct. Mater..

[15] Corbet C.M., Sonde S.S., Tutuc E., Banerjee S.K. (2016). Improved Contact Resistance in ReSe_2_ Thin Film Field-Effect Transistors. Appl. Phys. Lett..

[16] Khan M.F., Rehman S., Akhtar I., Aftab S., Ajmal H.M.S., Khan W., Kim D., Eom J. (2019). High Mobility ReSe_2_ Field Effect Transistors: Schottky-Barrier-Height-Dependent Photoresponsivity and Broadband Light Detection with Co Decoration. 2D Mater..

[17] Xing L., Yan X., Zheng J., Xu G., Lu Z., Liu L., Wang J., Wang P., Pan X., Jiao L. (2019). Highly Crystalline ReSe_2_ Atomic Layers Synthesized by Chemical Vapor Transport. InfoMat.

[18] Tian X., Liu Y. (2021). Van Der Waals Heterojunction ReSe_2_/WSe_2_ Polarization-Resolved Photodetector. J. Semicond..

[19] Oliva R., Laurien M., Dybala F., Kopaczek J., Qin Y., Tongay S., Rubel O., Kudrawiec R. (2019). Pressure Dependence of Direct Optical Transitions in ReS_2_ and ReSe_2_. Npj 2D Mater. Appl..

[20] Tongay S., Sahin H., Ko C., Luce A., Fan W., Liu K., Zhou J., Huang Y.-S., Ho C.-H., Yan J. (2014). Monolayer Behavior in Bulk ReS_2_ Due to Electronic and Vibrational Decoupling. Nat. Commun..

[21] Di Bartolomeo A., Urban F., Passacantando M., McEvoy N., Peters L., Iemmo L., Luongo G., Romeo F., Giubileo F. (2019). A WSe_2_ Vertical Field Emission Transistor. Nanoscale.

[22] Urban F., Martucciello N., Peters L., McEvoy N., Di Bartolomeo A. (2018). Environmental Effects on the Electrical Characteristics of Back-Gated WSe_2_ Field-Effect Transistors. Nanomaterials.

[23] Grillo A., Di Bartolomeo A. (2021). A Current–Voltage Model for Double Schottky Barrier Devices. Adv. Electron. Mater..

[24] Di Bartolomeo A., Grillo A., Urban F., Iemmo L., Giubileo F., Luongo G., Amato G., Croin L., Sun L., Liang S.-J. (2018). Asymmetric Schottky Contacts in Bilayer MoS_2_ Field Effect Transistors. Adv. Funct. Mater..

[25] Ezhilmaran B., Patra A., Benny S., Sreelakshmi M.R., Akshay V.V., Bhat S.V., Rout C.S. (2021). Recent Developments in the Photodetector Applications of Schottky Diodes Based on 2D Materials. J. Mater. Chem. C.

[26] Giubileo F., Di Bartolomeo A. (2017). The Role of Contact Resistance in Graphene Field-Effect Devices. Prog. Surf. Sci..

[27] Pelella A., Grillo A., Urban F., Giubileo F., Passacantando M., Pollmann E., Sleziona S., Schleberger M., Di Bartolomeo A. (2021). Gate-Controlled Field Emission Current from MoS_2_ Nanosheets. Adv. Electron. Mater..

[28] Di Bartolomeo A., Pelella A., Urban F., Grillo A., Iemmo L., Passacantando M., Liu X., Giubileo F. (2020). Field Emission in Ultrathin PdSe_2_ Back-Gated Transistors. Adv. Electron. Mater..

[29] Sun J., Passacantando M., Palummo M., Nardone M., Kaasbjerg K., Grillo A., Di Bartolomeo A., Caridad J.M., Camilli L. (2020). Impact of Impurities on the Electrical Conduction of Anisotropic Two-Dimensional Materials. Phys. Rev. Appl..

[30] Di Bartolomeo A., Urban F., Pelella A., Grillo A., Iemmo L., Faella E., Giubileo F. Electrical Transport in Two-Dimensional PdSe2 and Mos2 Nanosheets. Proceedings of the 2020 IEEE 20th International Conference on Nanotechnology (IEEE-NANO).

[31] Kang B., Kim Y., Cho J.H., Lee C. (2017). Ambipolar Transport Based on CVD-Synthesized ReSe_2_. 2D Mater..

[32] Urban F., Lupina G., Grillo A., Martucciello N., Di Bartolomeo A. (2020). Contact Resistance and Mobility in Back-Gate Graphene Transistors. Nano Express.

[33] Pelella A., Kharsah O., Grillo A., Urban F., Passacantando M., Giubileo F., Iemmo L., Sleziona S., Pollmann E., Madauß L. (2020). Electron Irradiation of Metal Contacts in Monolayer MoS_2_ Field-Effect Transistors. ACS Appl. Mater. Interfaces.

[34] Di Bartolomeo A., Genovese L., Giubileo F., Iemmo L., Luongo G., Foller T., Schleberger M. (2017). Hysteresis in the Transfer Characteristics of MoS_2_ Transistors. 2D Mater..

[35] Giubileo F., Iemmo L., Passacantando M., Urban F., Luongo G., Sun L., Amato G., Enrico E., Di Bartolomeo A. (2019). Effect of Electron Irradiation on the Transport and Field Emission Properties of Few-Layer MoS_2_ Field-Effect Transistors. J. Phys. Chem. C.

[36] Sangwan V.K., Jariwala D., Kim I.S., Chen K.-S., Marks T.J., Lauhon L.J., Hersam M.C. (2015). Gate-Tunable Memristive Phenomena Mediated by Grain Boundaries in Single-Layer MoS_2_. Nat. Nanotechnol..

[37] Di Bartolomeo A., Pelella A., Liu X., Miao F., Passacantando M., Giubileo F., Grillo A., Iemmo L., Urban F., Liang S. (2019). Pressure-Tunable Ambipolar Conduction and Hysteresis in Thin Palladium Diselenide Field Effect Transistors. Adv. Funct. Mater..

[38] Urban F., Giubileo F., Grillo A., Iemmo L., Luongo G., Passacantando M., Foller T., Madauß L., Pollmann E., Geller M.P. (2019). Gas Dependent Hysteresis in MoS_2_ Field Effect Transistors. 2D Mater..

[39] Lee C., Rathi S., Khan M.A., Lim D., Kim Y., Yun S.J., Youn D.-H., Watanabe K., Taniguchi T., Kim G.-H. (2018). Comparison of Trapped Charges and Hysteresis Behavior in HBN Encapsulated Single MoS_2_ Flake Based Field Effect Transistors on SiO_2_ and HBN Substrates. Nanotechnology.

[40] Knobloch T., Rzepa G., Illarionov Y.Y., Waltl M., Schanovsky F., Stampfer B., Furchi M.M., Mueller T., Grasser T. (2018). A Physical Model for the Hysteresis in MoS_2_ Transistors. IEEE J. Electron Devices Soc..

[41] Shu J., Wu G., Guo Y., Liu B., Wei X., Chen Q. (2016). The Intrinsic Origin of Hysteresis in MoS_2_ Field Effect Transistors. Nanoscale.

[42] Silva B., Rodrigues J., Sompalle B., Liao C.-D., Nicoara N., Borme J., Cerqueira F., Claro M., Sadewasser S., Alpuim P. (2021). Efficient ReSe_2_ Photodetectors with CVD Single-Crystal Graphene Contacts. Nanomaterials.

[43] Han Y., Zheng X., Fu M., Pan D., Li X., Guo Y., Zhao J., Chen Q. (2016). Negative Photoconductivity of InAs Nanowires. Phys. Chem. Chem. Phys..

[44] Di Bartolomeo A., Urban F., Faella E., Grillo A., Pelella A., Giubileo F., Askari M.B., McEvoy N., Gity F., Hurley P.K. (2021). PtSe_2_ Phototransistors with Negative Photoconductivity. J. Phys. Conf. Ser..

[45] Urban F., Gity F., Hurley P.K., McEvoy N., Di Bartolomeo A. (2020). Isotropic Conduction and Negative Photoconduction in Ultrathin PtSe_2_ Films. Appl. Phys. Lett..

[46] Grillo A., Faella E., Pelella A., Giubileo F., Ansari L., Gity F., Hurley P.K., McEvoy N., Di Bartolomeo A. (2021). Coexistence of Negative and Positive Photoconductivity in Few-Layer PtSe_2_ Field-Effect Transistors. Adv. Funct. Mater..

[47] Cui B., Xing Y., Han J., Lv W., Lv W., Lei T., Zhang Y., Ma H., Zeng Z., Zhang B. (2021). Negative Photoconductivity in Low-Dimensional Materials. Chin. Phys. B.

